# Interpretable Machine Learning for LPR Risk Estimation: A Single‐Center Retrospective Case–Control Study

**DOI:** 10.1002/jgh3.70404

**Published:** 2026-04-14

**Authors:** Chunrou Long, Yuan Li, Xiaoxue Zhang, Jianhui Li, Xin Hao, Haiyang Hua

**Affiliations:** ^1^ Chengde Medical University Chengde Hebei China; ^2^ Department of Gastroenterology Chengde Central Hospital Chengde Hebei China

**Keywords:** endoscopy, interpretability, laryngopharyngeal reflux, machine learning, risk prediction

## Abstract

**Objective:**

Based on RSI and RFS scores, an interpretable machine learning model was constructed to identify risk factors for LPR, aiming to screen high‐risk patients requiring 24‐h pH‐impedance monitoring and provide reference for clinical decision‐making.

**Methods:**

A retrospective case–control study included 537 patients who underwent painless gastroscopy (June 2024–June 2025), split into training (*n* = 376) and validation (*n* = 161) sets at 7:3. Nested cross‐validation‐based Boruta algorithm screened key predictors. Nine machine learning models were built, with performance evaluated via F1 score, recall, accuracy, precision, AUC, and Brier score in the validation set. AUC stability was validated by 1000 Bootstrap resamplings. Decision curve analysis assessed clinical net benefit, SHAP method interpreted the optimal model, and a web‐based risk calculator was developed.

**Results:**

Six independent LPR predictors were identified: arytenoid IPCL dilation, abdominal circumference, reflux esophagitis, alcohol consumption history, right lateral sleeping position, and GEFV grade III/IV. The random forest model performed best (F1 = 0.725, recall = 0.716, accuracy = 0.727, precision = 0.734), with Bootstrap‐validated AUC of 0.815 (95% CI: 0.753–0.873). Calibration curve showed good fit, decision curve analysis confirmed clinical net benefit across thresholds. SHAP analysis ranked feature contributions as: arytenoid IPCL dilation, abdominal circumference, reflux esophagitis, alcohol consumption history, right lateral sleeping position, GEFV grade III/IV. A web‐based calculator was deployed (URL: http://127.0.0.1:7292).

**Conclusion:**

This study constructed and validated an interpretable machine learning model integrating endoscopic and clinical indicators. The model demonstrates good discriminative ability and calibration and can serve as an auxiliary screening tool for patients with suspected LPR to help clinicians identify high‐risk individuals who require priority 24‐h MII‐pH monitoring.

## 1 Introduction

Laryngopharyngeal reflux (LPR) refers to the backflow of gastroduodenal contents into the laryngopharyngeal region, causing a series of symptoms such as foreign body sensation, dysphonia, and dysphagia [1, 2]. Its incidence in China has been increasing year by year [3]. Long‐term stimulation by refluxate can lead to chronic inflammation, mucosal hyperplasia, and even progression to laryngopharyngeal neoplasms [4]. As LPR symptoms (e.g., chronic cough, hoarseness, and throat irritation) are often non‐specific and may involve multiple disciplines such as dentistry, otorhinolaryngology, respiratory medicine, and gastroenterology, there is currently a lack of unified diagnostic criteria and standardized operational procedures. With the widespread adoption of painless gastroscopy, endoscopists have the opportunity to systematically observe the laryngopharyngeal mucosa while examining the esophagus, stomach, and duodenum. This presents a novel opportunity to integrate endoscopic features with clinical indicators for building an LPR risk assessment system.

Machine learning (ML), with its powerful capabilities in data mining, classification, and prediction, has been widely applied in the medical field [5, 6]. However, traditional algorithms often rely on statistical features and lack interpretability [7]. SHapley Additive exPlanations (SHAP), a post hoc interpretability algorithm based on the Shapley value from game theory, quantifies the impact of each feature on the model output by calculating its average marginal contribution across all possible feature subsets [8]. SHAP not only ensures the fairness and consistency of explanations but also reveals interaction mechanisms between features, providing a reliable theoretical foundation for model predictions. Accordingly, this study aims to provide a risk stratification reference for patients with suspected LPR based on symptom scores and to identify high‐risk individuals who require priority 24‐h MII‐pH monitoring.

## 2 Materials and Methods

### 2.1 Clinical Data and Grouping

A case–control study design was employed. A total of 537 patients who underwent painless gastroscopy at our institution between June 2024 and June 2025 were retrospectively enrolled. Inclusion criteria: (1) Age between 18 and 75 years; (2) LPR Group: Reflux Symptom Index (RSI) > 13 and Reflux Finding Score (RFS) > 7 [9]; (3) Non‐LPR Control Group: RSI ≤ 13 and RFS ≤ 7, and absence of typical gastroesophageal reflux disease (GERD) symptoms (heartburn and acid regurgitation). Exclusion criteria: (1) History of acute upper respiratory tract infection or pharyngolaryngitis within 4 weeks prior to examination; (2) Presence of laryngopharyngeal space‐occupying lesions, surgical history, or significant structural abnormalities; (3) Presence of acute inflammation or bleeding in the upper gastrointestinal tract, esophageal/gastric varices, tumors, or history of gastroesophageal surgery; (4) Image quality insufficient for analysis; (5) Incomplete clinical data or inability to cooperate in completing the questionnaire. Ultimately, 537 patients were included, comprising 239 in the LPR group and 298 in the non‐LPR control group. The study protocol was approved by the Institutional Ethics Committee (Approval No. CDCHLL2023‐460), and written informed consent was obtained from all participants.

### 2.2 Clinical and Endoscopic Parameters

#### 2.2.1 General Clinical Data

General clinical data were collected, including age, sex, height, weight, abdominal circumference, smoking history, alcohol consumption history, dietary preferences (preference for greasy foods, preference for strong tea), sleeping position (right lateral decubitus position [RLDP; yes/no]), constipation, history of calcium channel blocker use, hyperlipidemia, hyperuricemia, hypertension, history of calcium antagonist use, and type 2 diabetes mellitus. The definitional criteria for related indicators in this study are as follows: Smoking: Sustained or cumulative smoking of ≥ 1 cigarette per day for ≥ 6 months [10]. Alcohol Consumption: For males: consuming ≥ 25 g of alcohol per occasion and drinking ≥ 3 times per week; for females: consuming ≥ 15 g of alcohol per occasion and drinking ≥ 3 times per week. The drinking history must be continuous or cumulative for ≥ 6 months [11]. Constipation: Meeting the Rome IV diagnostic criteria (including symptoms such as difficulty defecating, sensation of incomplete evacuation, and bowel movement frequency < 3 times per week) [12]. Definition of Dietary Preferences: Preference for Greasy Foods: Meeting any one of the following conditions: ① Consuming fried foods (e.g., fried chicken and fried gluten) ≥ 3 times per week; ② Daily cooking oil consumption significantly exceeding the recommended amount (≥ 50 g per person per day); ③ Preference for high‐fat foods (e.g., fatty meat and animal organs) and consumption ≥ 3 times per week. Preference for Strong Tea: Drinking strong tea (≥ 10 g of tea leaves per day or tea with a dark brown color and strong bitterness) ≥ 1 time per day, sustained for ≥ 6 months. Preference for Right Lateral Decubitus Position: Patients reported that for ≥ 15 days within the past 30 nights, the right lateral decubitus position was their primary sleeping posture. Abdominal Circumference Measurement: Measured as per our hospital's routine nursing protocol. Measurement was performed in the morning under fasting conditions. A nurse used a non‐elastic soft tape to measure at the midpoint level between the iliac crest and the lower rib margin at the end of expiration, ensuring the tape was snug against the skin without compressing soft tissue.

#### 2.2.2 Endoscopic Indicators

**FIGURE 1:**
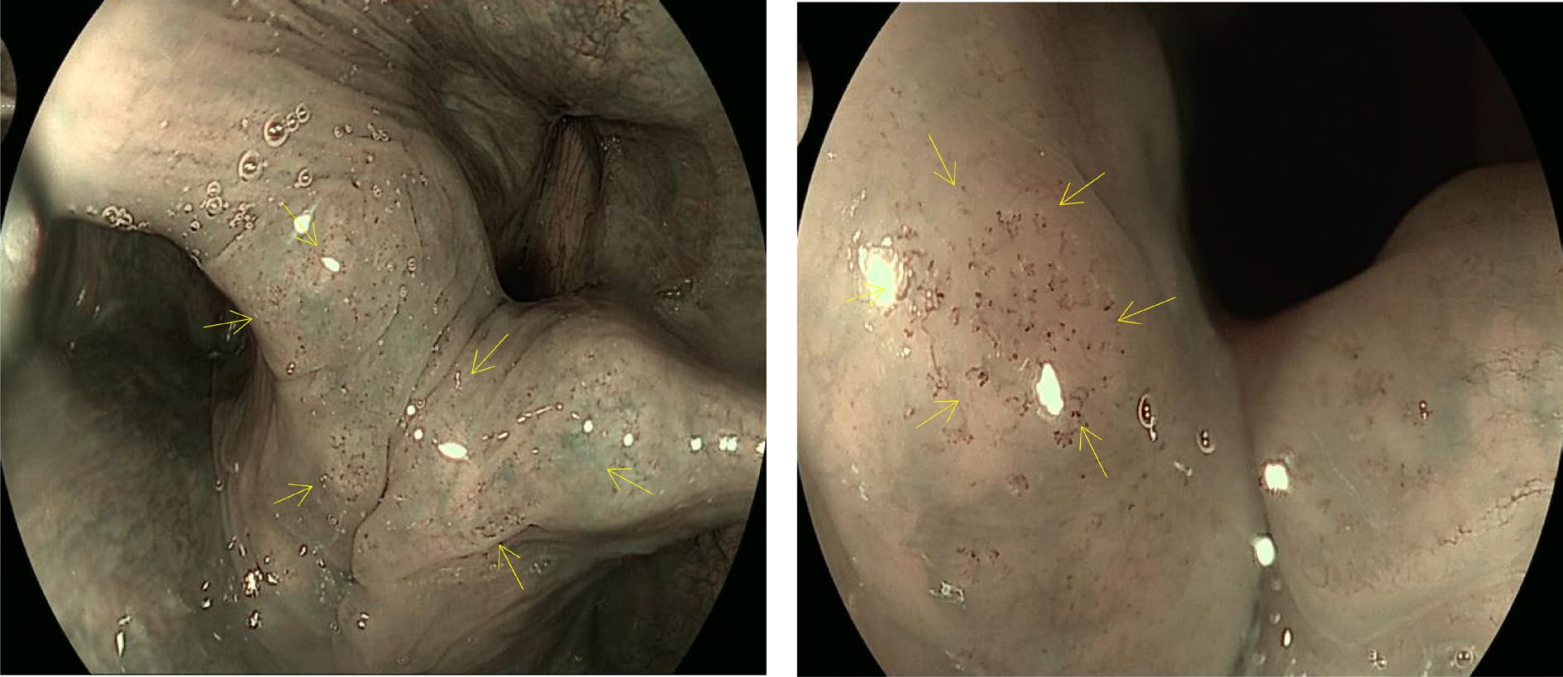
Typical endoscopic images of arytenoid IPCL dilation.

The following endoscopic findings were systematically recorded: presence of arytenoid IPCL dilation, ectopic gastric mucosa in the esophagus, reflux esophagitis, hiatal hernia, and Hill grade of the gastroesophageal flap valve [13]. As current guidelines and literature lack a clear definition for “arytenoid IPCL dilation” this study defined it as: under endoscopy, the presence of punctate, tubular, or reticular dilated intrapapillary capillary loops (IPCLs) in the arytenoid region, interarytenoid region, or postcricoid region (see Figure 1). To assess the reproducibility of this endoscopic sign, 50 images from the study (25 positive and 25 negative) were randomly selected. Two senior endoscopists (both with over 10 years of experience in digestive endoscopy and not involved in initial data collection for this study) independently evaluated the images based on the provided definition under blinded conditions. Inter‐observer agreement was assessed using Cohen's Kappa coefficient, yielding a Kappa value of 0.78 (95% CI: 0.65–0.91). Observer A re‐evaluated the same image set 1 week later, and intra‐observer agreement was calculated as a Kappa value of 0.85 (95% CI: 0.74–0.96).

### 2.3 Data Processing

#### 2.3.1 Feature Selection for LPR Risk Using Nested Cross‐Validation With Boruta Algorithm

The dataset was randomly divided into a training set (*n* = 376) and a validation set (*n* = 161) in a 7:3 ratio using stratified sampling. Within the training set, a nested structure of “outer 10‐fold cross‐validation with inner Boruta screening” was employed. In each fold of the outer loop, the training set was further split into an outer training subset and an outer validation subset. The inner loop independently ran the Boruta algorithm based solely on the outer training subset of that fold. Boruta generates shadow features via random forest and classifies original variables as “Confirmed Important” (green), “Tentative” (yellow), or “Rejected” (red) based on Z‐scores of feature importance and statistical significance. The feature selection process in each fold was completely independent, with results not shared across folds. To determine the final feature set, we calculated the frequency with which each feature was classified as “Confirmed Important” (green) across all folds. Features that were consistently confirmed as important in ≥ 5 folds (i.e., ≥ 50% of folds) were identified as definitive LPR risk predictors.

#### 2.3.2 Machine Learning Model Selection and Evaluation

Nine machine learning models were constructed using the R caret package, based on the selected risk features: Logistic Regression (Logistic), Random Forest (RF), Support Vector Machine (SVM), Gradient Boosting Machine (GBM), Extreme Gradient Boosting (XGBoost), Neural Network (NeuralNet), k‐Nearest Neighbors (KNN), Elastic Net (ElasticNet), and Linear Discriminant Analysis (LDA). Model training and hyperparameter tuning were performed on the training set using 10‐fold cross‐validation repeated 3 times. The tuning process utilized only the final feature set obtained from the nested Boruta selection. Model performance was evaluated using the F1 score, area under the receiver operating characteristic curve (AUC), Brier score, recall, precision, and accuracy. The robustness of the AUC was further validated via 1000 bootstrap resampling iterations. Model calibration was assessed using calibration curves with 10 equal‐width bins. Given the clinical priority of sensitivity in LPR screening tasks, model selection primarily considered the comprehensive performance on the independent validation set, focusing on AUC, recall, and F1 score to identify the optimal candidate model. To enhance the reliability of predicted probabilities, Platt scaling was applied for probability calibration of the optimal candidate model. A logistic regression calibrator was fitted using the predicted probabilities from the training set as input, and this calibrator was then applied to correct the predicted probabilities of the validation set. The calibrated probabilities were used to generate calibration curves, calculate the Brier score, and were integrated into subsequent decision curve analysis and the interactive calculator, ensuring the accuracy and practicality of clinical risk assessment.

#### 2.3.3 Interpreting and Visualizing the Selected Machine Learning Model Using SHAP

This study employed SHAP (SHapley Additive exPlanations) analysis to elucidate the decision‐making mechanism of the machine learning model. All SHAP analyses were conducted on the independent validation set. Feature importance was quantified by the mean absolute SHAP value (|SHAP|), where a larger value indicates a greater contribution to LPR risk prediction. The results were visualized using an ordered bar plot. The directional effect of a feature was determined by the sign of its SHAP value: a positive value represents a risk‐enhancing effect, while a negative value indicates a protective effect.

#### 2.3.4 Development of a Web Calculator for the Selected Machine Learning Model

An interactive model interpretation platform was developed using the R language, enabling the visualization of the machine learning model's interpretations and its local deployment.

### 2.4 Statistical Methods

All analyses were performed using R (version 4.4.3) and SPSS Statistics (version 26.0). Continuous variables were assessed for normality using the Shapiro–Wilk test and are described as follows: normally distributed data are presented as mean ± standard deviation (x̄ ± SD), with between‐group comparisons made using the independent samples *t*‐test; non‐normally distributed data are presented as median [interquartile range] (M[Q1 − Q3]), with comparisons made using the Mann–Whitney *U* test. Categorical variables are reported as counts (percentages) [*n* (%)] and were analyzed using the chi‐square test. All tests were two‐tailed, with a significance threshold (*α*) set at 0.05.

## 3 Results

### 3.1 Comparison of Clinical Data Between the Training and Validation Sets

This study included a total of 537 patients, who were randomly allocated into a training set (*n* = 376) and a validation set (*n* = 161). Comparison of the baseline characteristics between the two sets revealed no statistically significant differences for any variable (all *p* values > 0.05). Additionally, all standardized mean differences were less than 0.2 (Table 1), indicating that the populations in the two sets were balanced and comparable.

**TABLE 1:** Baseline characteristics of the study cohorts.

Variable	Level	Overall	Train	Validation	*p*	SMD
*n*		537	376	161		
Age		55.00 [46.00, 62.00]	55.00 [45.75, 62.00]	56.00 [48.00, 62.00]	0.601	0.059
BMI media		24.61 [22.31, 26.85]	24.36 [22.13, 26.84]	25.10 [23.29, 27.34]	0.052	0.159
Waistline		79.00 [73.00, 85.00]	79.00 [73.00, 84.00]	79.00 [73.30, 85.00]	0.799	0.024
Sex	Male	253 (47.1)	175 (46.5)	78 (48.4)	0.707	0.038
	Female	284 (52.9)	201 (53.5)	83 (51.6)		
Smoking	No	438 (81.6)	303 (80.6)	135 (83.9)	0.398	0.085
	Yes	99 (18.4)	73 (19.4)	26 (16.1)		
Drinking_alcohol	No	347 (64.6)	238 (63.3)	109 (67.7)	0.375	0.093
	Yes	190 (35.4)	138 (36.7)	52 (32.3)		
Drink tea	No	452 (84.2)	315 (83.8)	137 (85.1)	0.797	0.036
	Yes	85 (15.8)	61 (16.2)	24 (14.9)		
RLDP	No	388 (72.3)	263 (69.9)	125 (77.6)	0.074	0.176
	Yes	149 (27.7)	113 (30.1)	36 (22.4)		
GFP	No	424 (79.0)	293 (77.9)	131 (81.4)	0.419	0.086
	Yes	113 (21.0)	83 (22.1)	30 (18.6)		
T2DM	No	495 (92.2)	351 (93.4)	144 (89.4)	0.159	0.140
	Yes	42 (7.8)	25 (6.6)	17 (10.6)		
CCB	No	461 (85.8)	326 (86.7)	135 (83.9)	0.418	0.081
	Yes	76 (14.2)	50 (13.3)	26 (16.1)		
HH	No	535 (99.6)	374 (99.5)	161 (100.0)	1.000	0.103
	Yes	2 (0.4)	2 (0.5)	0 (0.0)		
RE	No	335 (62.4)	232 (61.7)	103 (64.0)	0.629	0.047
	Yes	202 (37.6)	144 (38.3)	58 (36.0)		
Constipation	No	486 (90.5)	342 (91.0)	144 (89.4)	0.630	0.051
	Yes	51 (9.5)	34 (9.0)	17 (10.6)		
UA	No	505 (94.0)	351 (93.4)	154 (95.7)	0.426	0.101
	Yes	32 (6.0)	25 (6.6)	7 (4.3)		
HLD	No	427 (79.5)	299 (79.5)	128 (79.5)	1.000	< 0.001
	Yes	110 (20.5)	77 (20.5)	33 (20.5)		
A_IPCL_dilation	No	347 (64.6)	252 (67.0)	95 (59.0)	0.077	0.167
	Yes	190 (35.4)	124 (33.0)	66 (41.0)		
EGME	No	505 (94.0)	354 (94.1)	151 (93.8)	0.845	0.015
	Yes	32 (6.0)	22 (5.9)	10 (6.2)		
HT	No	384 (71.5)	267 (71.0)	117 (72.7)	0.755	0.037
	Yes	153 (28.5)	109 (29.0)	44 (27.3)		
GEFV	I/II	508 (94.6)	352 (93.6)	156 (96.9)	0.147	0.155
	III/IV	29 (5.4)	24 (6.4)	5 (3.1)		

Abbreviations: A‐IPCL dilation: dilation of capillary loops in the epithelial papillae of the arytenoid cartilage; CCB: history of calcium channel blocker use; EGME: esophagogastric mucosal ectopia; GEFV: gastroesophageal flap valve; GFP: preference for greasy food; HH: hiatal hernia; HLD: hyperlipidemia; HT: hypertension; RE: reflux esophagitis; RLDP: right lateral decubitus position; T2DM: Type 2 diabetes mellitus; UA: hyperuricemia.

### 3.2 Variable Selection and Model Development

#### 3.2.1 Risk Features for Laryngopharyngeal Reflux

**FIGURE 2:**
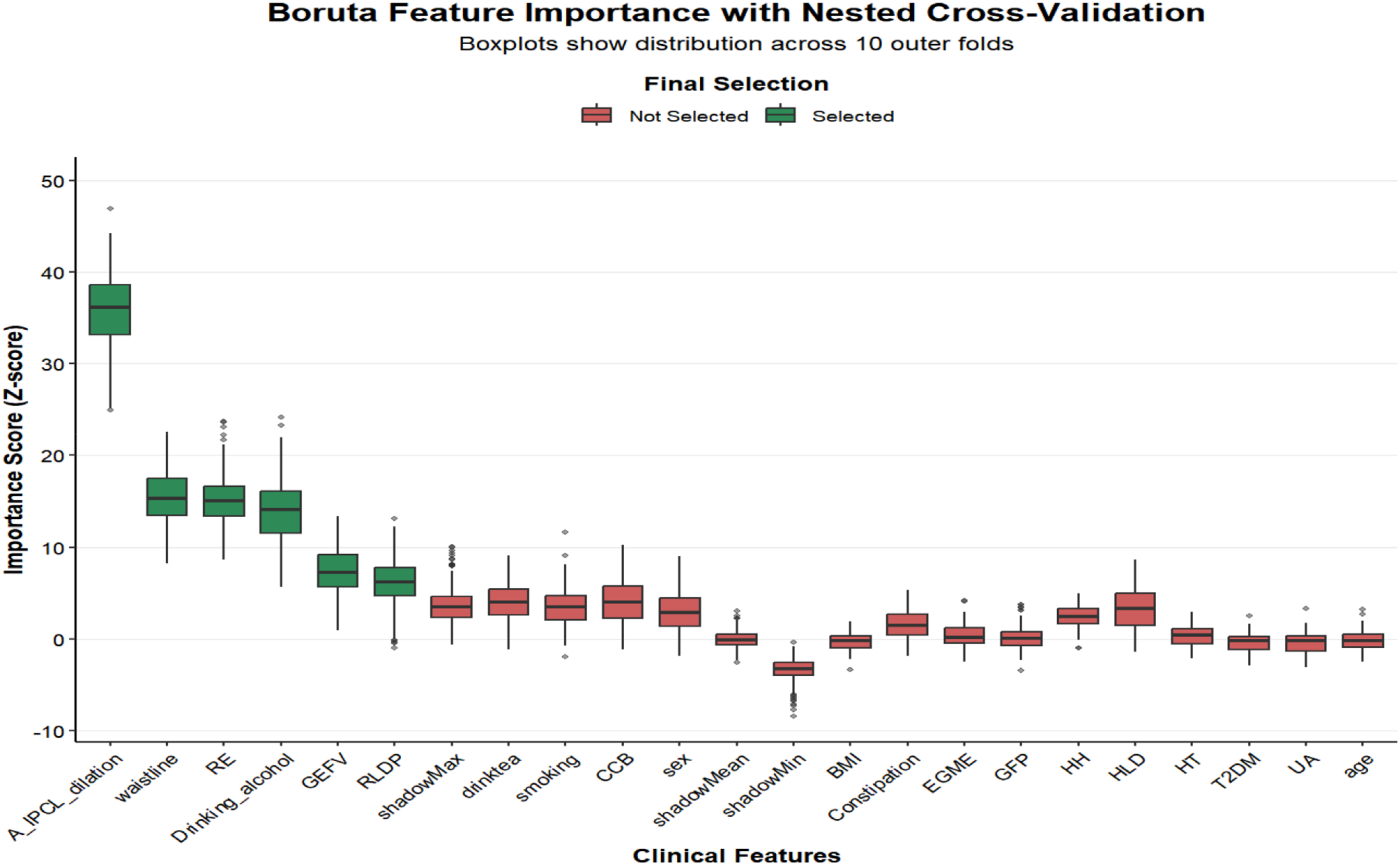
Box plot.

Utilizing the nested cross‐validation Boruta algorithm, six clinically significant risk factors for laryngopharyngeal reflux were identified: (1) arytenoid IPCL dilation, (2) increased abdominal circumference, (3) reflux esophagitis, (4) alcohol consumption, (5) gastroesophageal flap valve (GEFV) grade III/IV, and (6) sleeping in a right lateral decubitus position (Figure 2).

#### 3.2.2 Machine Learning Model Selection and Evaluation

**FIGURE 3:**
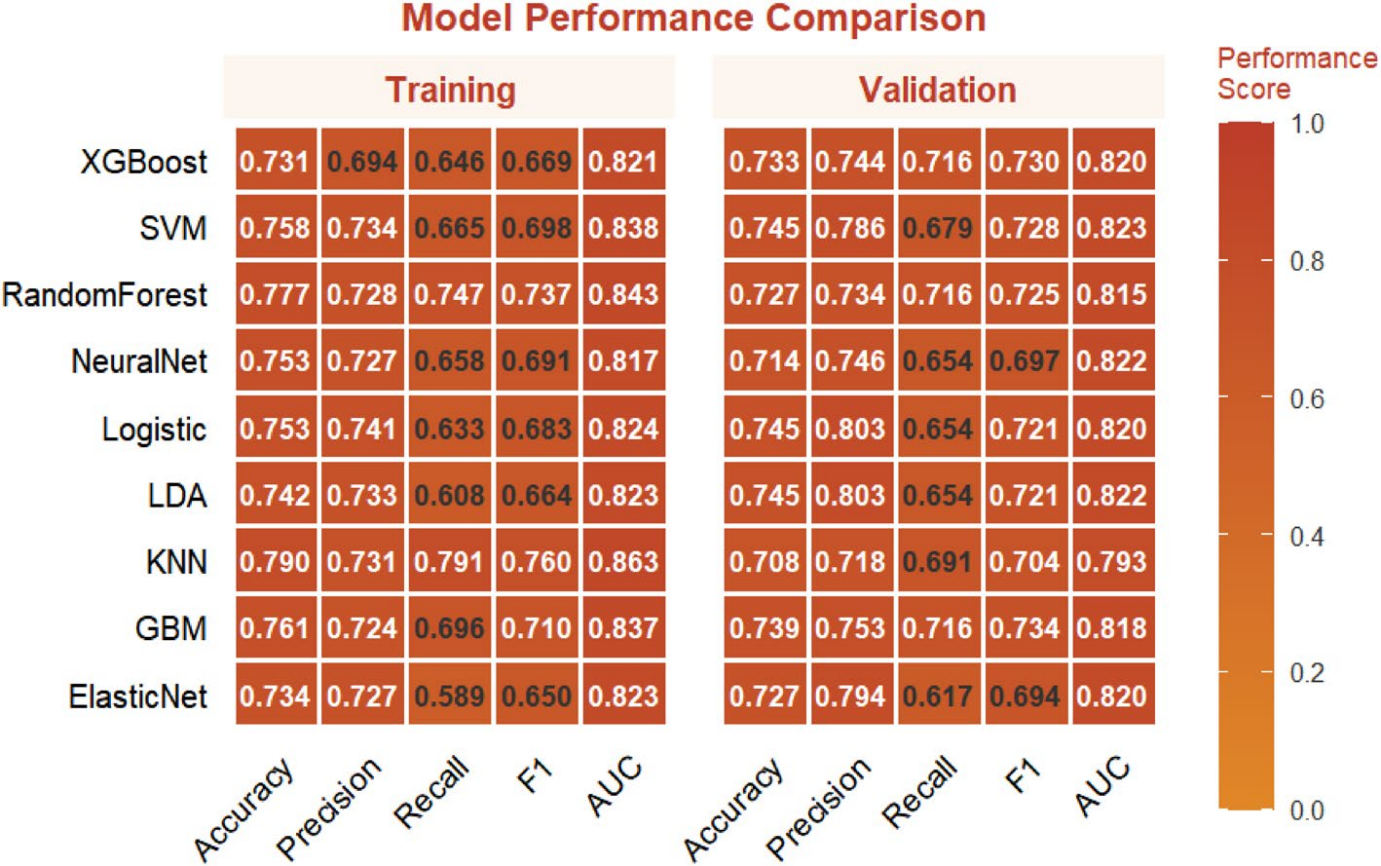
Heatmap for evaluating the performance of nine machine learning models.

**FIGURE 4:**
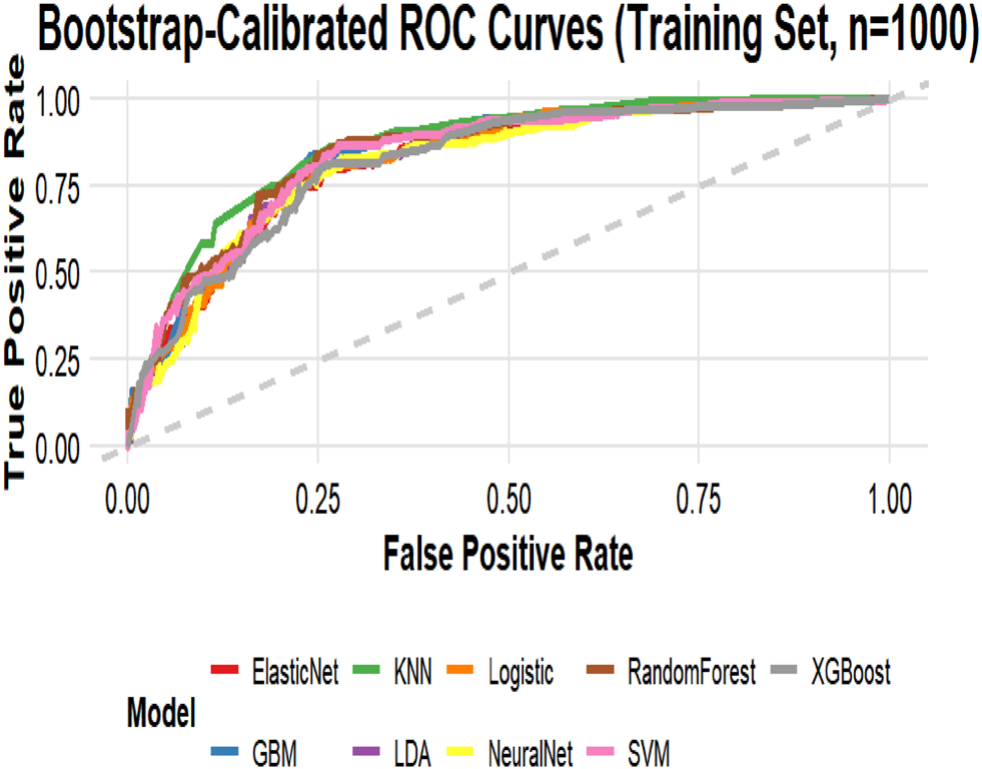
Bootstrap‐validated AUC values (1000 iterations) of the nine machine learning models for predicting laryngopharyngeal reflux in the training set.

**FIGURE 5:**
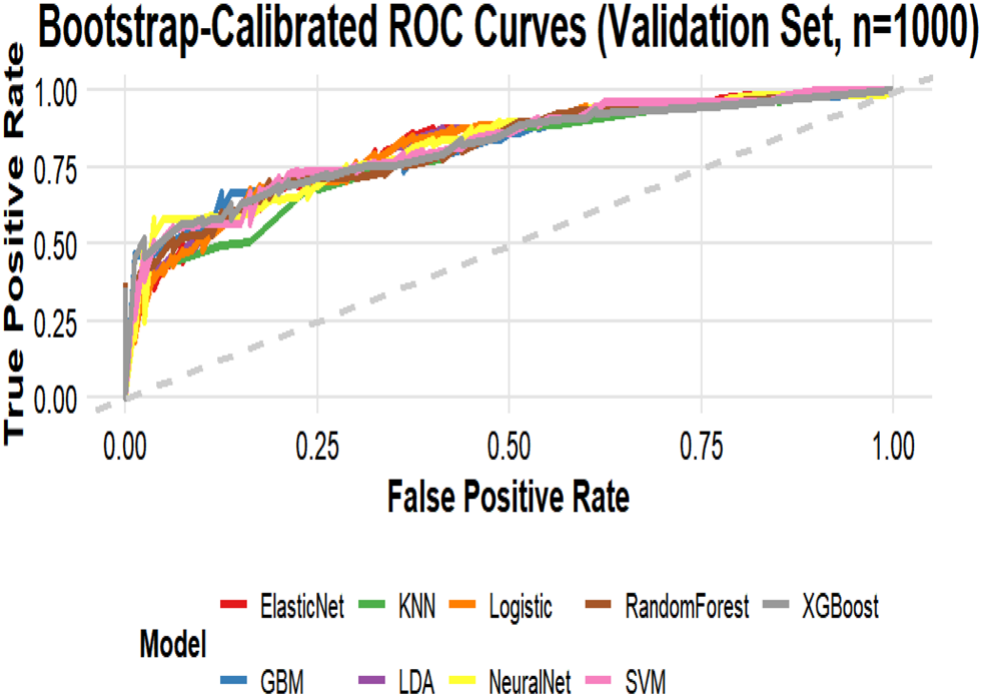
Bootstrap‐validated AUC values (1000 iterations) of the nine machine learning models for predicting laryngopharyngeal reflux in the validation set.

**FIGURE 6:**
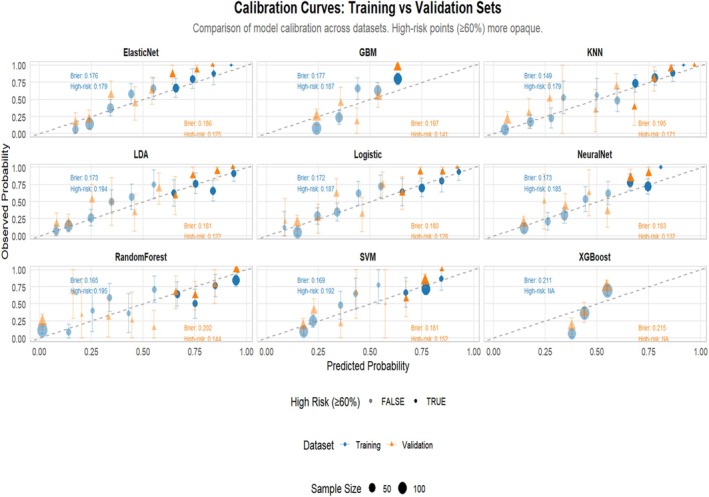
Calibration curves of nine machine learning models for laryngopharyngeal reflux prediction in both training and validation sets.

**FIGURE 7:**
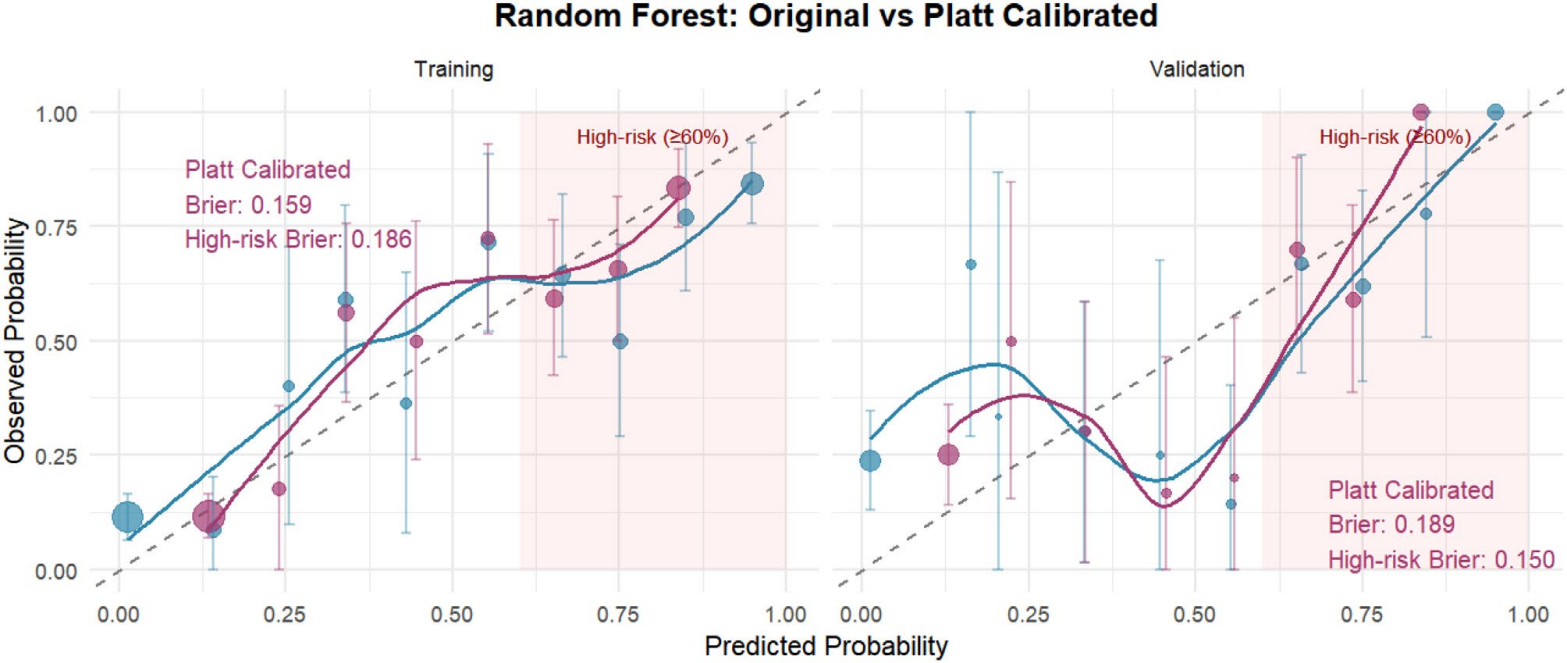
Calibration curve of the random forest model after Platt scaling.

**FIGURE 8:**
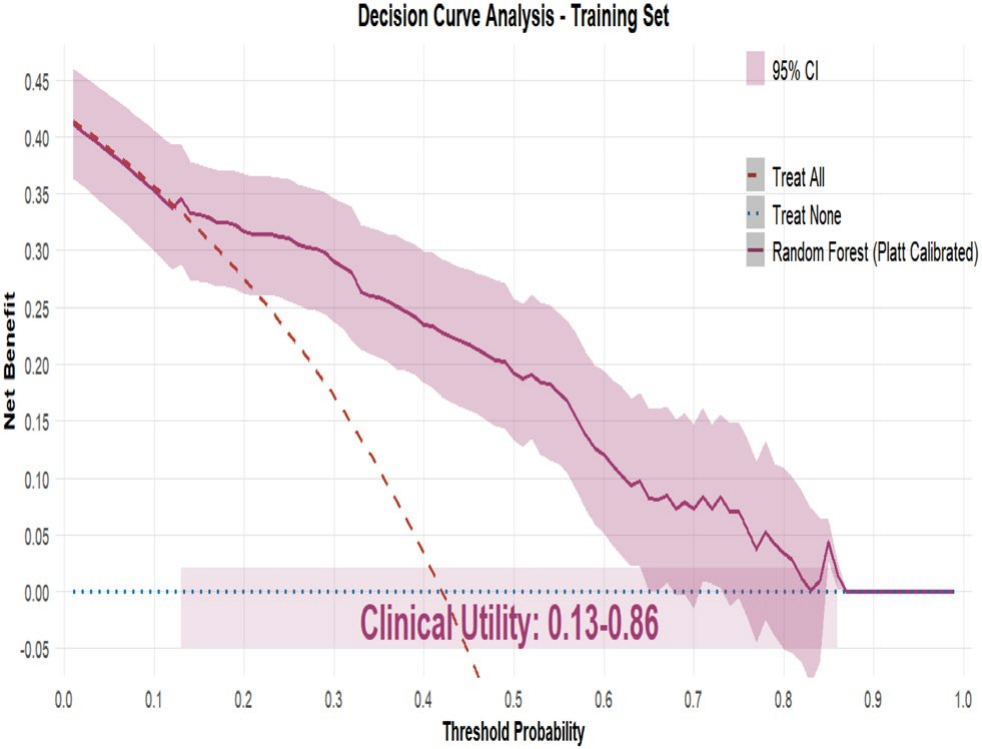
Decision curve analysis of the random forest model for predicting laryngopharyngeal reflux risk in the training set.

**FIGURE 9:**
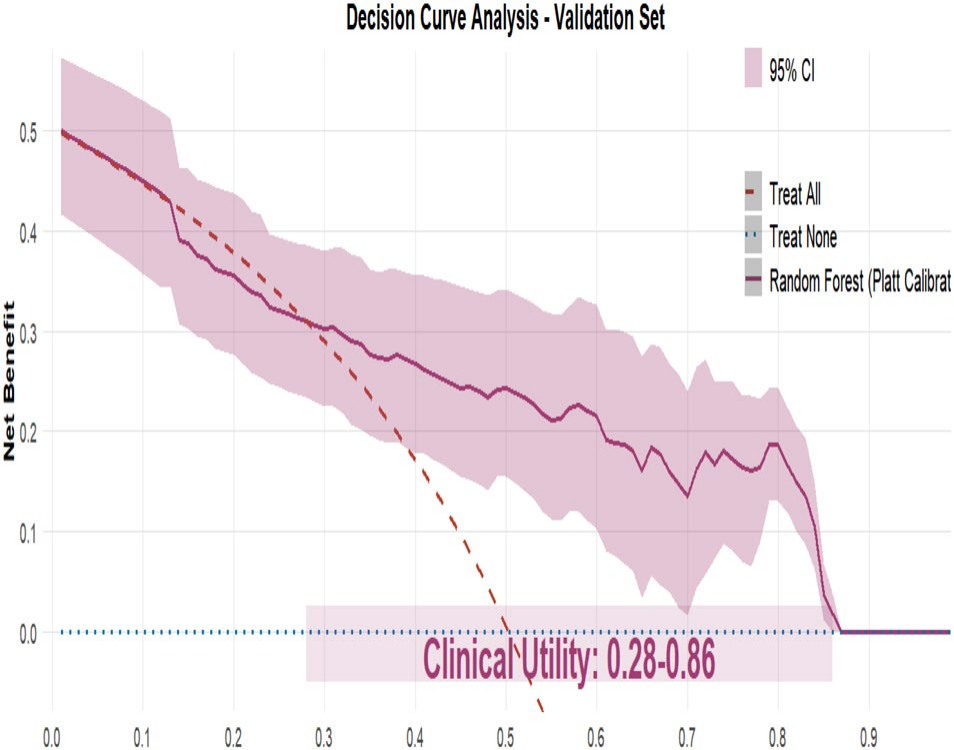
Decision curve analysis of the random forest model for predicting laryngopharyngeal reflux risk in the validation set.

Nine machine learning algorithms (Logistic Regression [LR], Random Forest [RF], Support Vector Machine [SVM], Gradient Boosting Machine [GBM], XGBoost, Neural Network, k‐Nearest Neighbors [KNN], Elastic Net, and Linear Discriminant Analysis [LDA]) were systematically compared using six performance metrics: F1 score, AUC, Brier score, recall, precision, and accuracy (Figure 3). Based on comprehensive discriminative performance, prioritizing AUC, recall, and F1 score on the validation set, the Random Forest model demonstrated the best performance. Bootstrap validation (1000 iterations) confirmed its robustness, yielding an AUC of 0.844 (95% CI: 0.805–0.882) on the training set and an AUC of 0.815 (95% CI: 0.753–0.873) on the validation set (Figures 4 and 5). The calibration curve indicated overall good calibration for the Random Forest model, with Brier scores of 0.165 for the training set and 0.202 for the validation set (Figure 6). After Platt scaling calibration, the Brier scores improved to 0.159 and 0.189 for the training and validation sets, respectively (Figure 7). Decision curve analysis (DCA) based on the calibrated probabilities showed that the Random Forest model provided a higher net benefit than both the “treat‐all” and “treat‐none” strategies across threshold probability ranges of 0.13–0.86 in the training set and 0.28–0.86 in the validation set, indicating good clinical utility (Figures 8 and 9).

#### 3.2.3 Interpreting and Visualizing the Random Forest Model Using the SHAP Method

**FIGURE 10:**
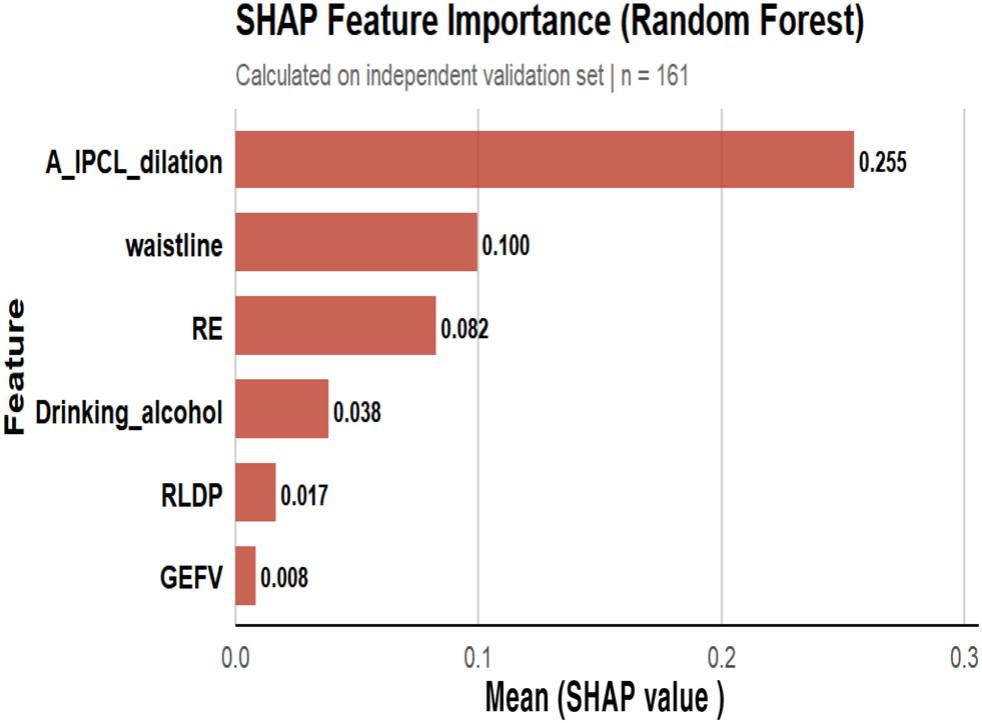
Bar plot.

**FIGURE 11:**
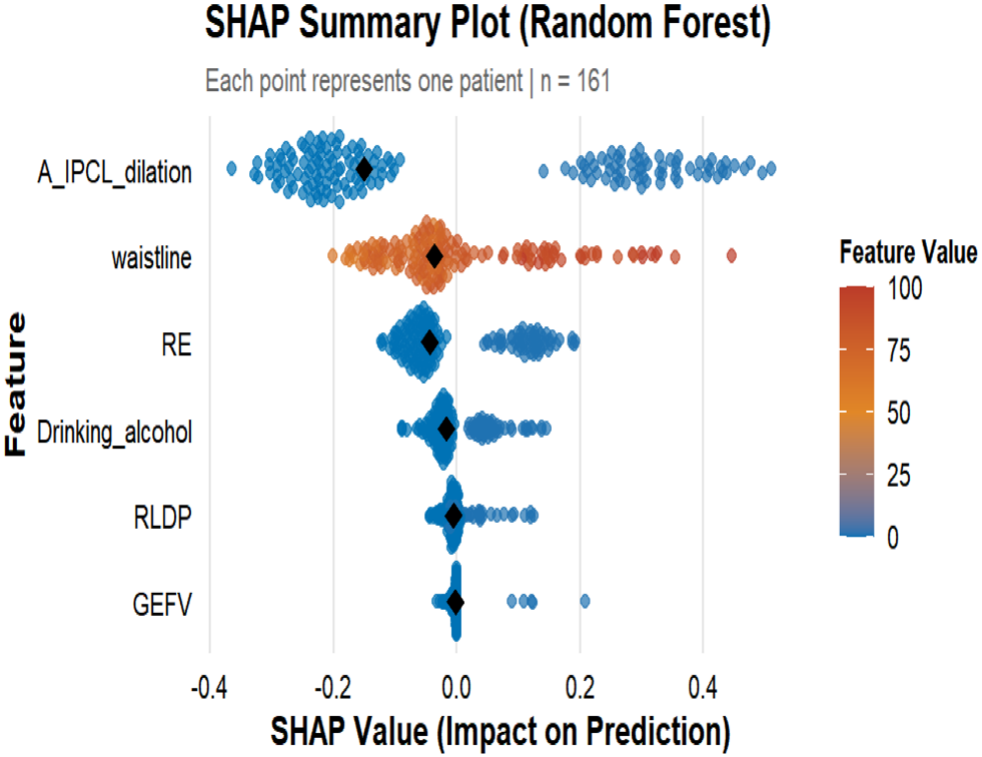
Summary plot.

SHAP value analysis (Figure 10) identified six clinically significant LPR risk predictors, ranked in descending order of importance: (1) arytenoid IPCL dilation (|SHAP| = 0.255), (2) abdominal circumference (|SHAP| = 0.100), (3) reflux esophagitis (|SHAP| = 0.082), (4) alcohol consumption (|SHAP| = 0.038), (5) right lateral sleeping position (|SHAP| = 0.017), and (6) gastroesophageal flap valve (GEFV) grade III/IV (|SHAP| = 0.008). The SHAP summary plot (Figure 11) demonstrated that these features predominantly exhibited a positive influence (SHAP value > 0) on LPR risk across the majority of samples.

#### 3.2.4 Web‐Based Calculator Developed From the Random Forest Model

**FIGURE 12:**
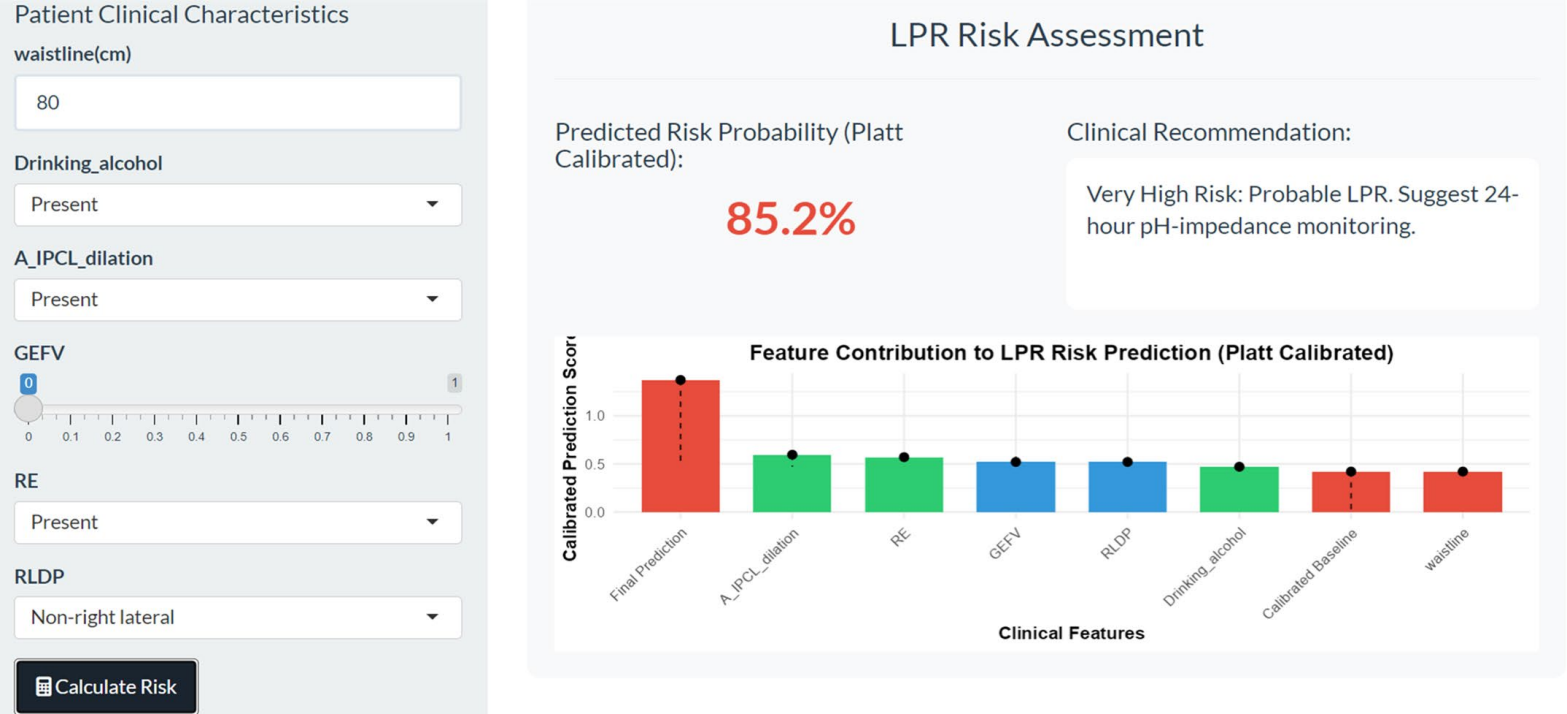
Interface for laryngopharyngeal reflux risk assessment based on Random Forest Mode.

A web‐based risk calculator (http://127.0.0.1:7292) was developed based on the optimal model and deployed on a local server, as illustrated in Figure 12.

## 4 Discussion

The prevalence of laryngopharyngeal reflux (LPR) in Chinese otolaryngology‐head and neck surgery outpatient clinics is as high as 10.15%. Its pathogenesis is related to, yet distinct from, gastroesophageal reflux disease (GERD) [3]. Unlike GERD, patients with LPR often lack typical symptoms such as acid regurgitation and heartburn. Instead, they frequently present with non‐specific manifestations like throat irritation, persistent throat clearing, hoarseness, and chronic cough as initial symptoms, which are easily confused with conditions such as chronic pharyngolaryngitis, allergic rhinitis, and asthma [1, 2]. This leads to high rates of missed diagnosis and misdiagnosis in clinical practice, significantly impairing patients' quality of life. Although 24‐h multichannel intraluminal impedance‐pH monitoring is considered the “gold standard,” it is an invasive procedure with high cost and limited accessibility, making it difficult to implement widely in primary care hospitals [14]. Against this backdrop, this study aimed to identify LPR risk factors and construct an interpretable LPR prediction model, intending to provide a reference for the clinical diagnosis and treatment of LPR.

The SHAP bar chart analysis in this study revealed that the key clinical features influencing LPR risk, ranked in descending order of importance, are: dilation of the IPCL in the arytenoid region, abdominal circumference, reflux esophagitis, alcohol consumption, right lateral sleeping position, and gastroesophageal flap valve (GEFV) grade III/IV. SHAP analysis demonstrated that these features predominantly exhibit a positive impact on LPR risk across the majority of samples. Beyond quantifying the contribution of each feature, SHAP analysis also identified instances where risk factors showed negative contributions in a minority of samples. This phenomenon likely reflects the heterogeneity of clinical disease. For example, while some patients may have risk factors such as alcohol consumption, the negative effect might be offset by protective factors not included in the model, such as strong esophageal motility, high upper esophageal sphincter pressure, or refluxate consisting primarily of non‐acidic components. Increased abdominal circumference promotes reflux through multiple mechanisms. On one hand, central obesity directly elevates intra‐abdominal and intragastric pressure, thereby impairing the anti‐reflux barrier function of the gastroesophageal junction (GEJ). On the other hand, the abnormal endocrine and metabolic activities of visceral adipose tissue can mediate a systemic low‐grade inflammatory state, and the pro‐inflammatory cytokines it releases have also been confirmed to be closely associated with the occurrence of reflux [15]. A study based on the South Korean adult population indicated that abdominal obesity is an independent risk factor for gastroesophageal reflux disease [16]. Subsequent Mendelian randomization analysis by Yuan S et al. further confirmed a significant causal association between central obesity and gastroesophageal reflux disease [17]. Laryngopharyngeal reflux (LPR) and gastroesophageal reflux disease (GERD) often coexist and interact. Research data show a high comorbidity rate: the incidence of GERD in LPR patients is 52.7%, while the incidence of LPR in GERD patients is 46.3% [18]. On the one hand, the severity of gastroesophageal reflux disease (GERD) is negatively correlated with upper esophageal sphincter (UES) pressure. More severe reflux esophagitis (RE) is associated with lower UES pressure and more impaired function, making it easier for refluxate to breach the barrier and ascend to the laryngopharynx [19, 20]. On the other hand, long‐term or severe RE can impair the peristaltic function of the esophageal body, weakening its clearance capacity for refluxate. This leads to prolonged and intensified reflux episodes and promotes their upward extension [21]. Alcohol consumption can affect the efficiency of synchronous esophageal contractions, significantly reduce lower esophageal sphincter (LES) pressure, and stimulate gastric acid secretion, thereby impairing gastric motility. This results in decreased gastric and esophageal function, increasing the risk of reflux material ascending to the pharynx [22, 23]. When a patient lies in the right lateral decubitus position, the anatomical position of the gastric fundus is higher than that of the esophagus, reversing the pressure gradient between intragastric pressure and LES pressure. This increases the risk of laryngopharyngeal reflux [24]. Consistent with our findings, Schuitenmaker et al., using 24‐h pH‐impedance monitoring, demonstrated that compared to the right lateral position, the left lateral position reduces acid exposure time and shortens acid clearance time [25]. The gastroesophageal flap valve (GEFV) is a functional structure observed during gastroscopy, consisting of a semicircular, 180° musculo‐mucosal fold extending from the angle of His at the gastroesophageal junction [26]. When intragastric pressure rises, this valve acts to help prevent the reflux of gastric contents into the esophagus. A higher Hill grade indicates poorer barrier function, leading to more frequent or larger‐volume reflux of gastric contents into the esophagus and even the laryngopharynx [27]. The Lyon Consensus notes that patients with definitive evidence of reflux have a higher prevalence of abnormal Hill grades [28]. The findings of this study indicate that dilation of the IPCL in the arytenoid region is a significant endoscopic predictor for LPR risk. This result aligns with previous research. The study by Zheng et al. noted that laryngopharyngeal reflux can manifest under electronic chromoendoscopy as scattered or clustered brownish spots in the arytenoid, interarytenoid, and postcricoid regions, which are considered to represent dilated and tortuous intrapapillary capillary loops. The reported sensitivity was 82.6% and specificity 81.2%, suggesting significant predictive value for laryngopharyngeal reflux [29]. A prospective study combining electronic chromoendoscopy with 24‐h multichannel intraluminal impedance‐pH monitoring found that brownish microvasculature in the postcricoid mucosa was a characteristic feature of LPR and could regress following acid‐suppressive therapy, indicating a potential association with reflux [30]. This may be attributed to persistent microtrauma inflicted by refluxate on the laryngopharyngeal mucosa, leading to localized microinflammatory responses and dilation/tortuosity of the intrapapillary capillary loops. Concurrently, cycles of mucosal damage and repair may involve hemosiderin deposition or pigmentary changes induced by inflammatory mediators, contributing to the characteristic brownish appearance. Furthermore, this study observed heterogeneity in the distribution pattern of arytenoid IPCL dilation: some patients exhibited it in the interarytenoid area and bilateral arytenoids, while others had it confined to the interarytenoid and postcricoid regions. This heterogeneity might be related to variations in the composition of the refluxate (e.g., acid, weakly acidic, or non‐acid reflux) and its aggressiveness. Additionally, factors such as the pattern, frequency, duration of reflux events, as well as the patient's disease course and symptom severity, could influence its formation and visibility.

In summary, this study initially constructed and validated an interpretable random forest model for predicting LPR risk. The model demonstrated good discriminative performance and calibration in the validation set, and decision curve analysis confirmed its potential clinical utility. However, several limitations should be acknowledged. First, constrained by clinical practicalities and the limited availability of the gold standard—24‐h multichannel intraluminal impedance‐pH monitoring—this study did not utilize this gold standard for group stratification. The diagnosis of LPR relied solely on the RSI and RFS scores, which may have introduced selection bias. Therefore, the applicability of this model should currently be strictly confined to patients with suspected LPR based on RSI/RFS criteria, serving as a preliminary screening and risk stratification tool in clinical practice to identify high‐risk individuals requiring priority gold standard testing among the broader population presenting with laryngopharyngeal symptoms, rather than as a replacement for the gold standard. Second, the inclusion of smoking and drinking histories was based on retrospective collection and dichotomous assignment, lacking continuous data support, which limited in‐depth analysis of the strength of association between these variables and LPR. Finally, the model's generalizability is limited as only internal validation was performed. Future research requires external validation, recalibration, and continuous optimization in prospective, multicenter, large‐scale cohorts using gold standard diagnostic methods. The integration of additional potential physiological protective factors is also necessary to ultimately facilitate its translational application in clinical practice.

## Funding

This work was supported by S&T Program of Chengde (202303A017 and 202501A005).

## Ethics Statement

This study was approved by Chengde Central Hospital's Ethics Committee (No. CDCHLL2023‐460). Written informed consent was obtained from all participants prior to the study.

## Consent

All authors have reviewed the final manuscript and consent to its publication.

## Conflicts of Interest

The authors declare no conflicts of interest.

## Data Availability

The data that support the findings of this study are available on request from the corresponding author. The data are not publicly available due to privacy or ethical restrictions.
